# Compact Finite Field Multiplication Processor Structure for Cryptographic Algorithms in IoT Devices with Limited Resources

**DOI:** 10.3390/s22062090

**Published:** 2022-03-08

**Authors:** Atef Ibrahim, Fayez Gebali

**Affiliations:** 1 Computer Engineering Department, College of Computer Engineering and Sciences, Prince Sattam Bin Abdulaziz University, Al-Kharj 16278, Saudi Arabia; 2 Electrical and Computer Engineering Department, University of Victroia, Victoria, BC V8P 5C2, Canada; fayez@uvic.ca

**Keywords:** IoT security, IoT applications, IoT devices, security of cyber-physical system, cryptographic processors, finite-field multipliers, processor arrays, ultra low-power devices, word-serial multipliers, cryptography

## Abstract

The rapid evolution of Internet of Things (IoT) applications, such as e-health and the smart ecosystem, has resulted in the emergence of numerous security flaws. Therefore, security protocols must be implemented among IoT network nodes to resist the majority of the emerging threats. As a result, IoT devices must adopt cryptographic algorithms such as public-key encryption and decryption. The cryptographic algorithms are computationally more complicated to be efficiently implemented on IoT devices due to their limited computing resources. The core operation of most cryptographic algorithms is the finite field multiplication operation, and concise implementation of this operation will have a significant impact on the cryptographic algorithm’s entire implementation. As a result, this paper mainly concentrates on developing a compact and efficient word-based serial-in/serial-out finite field multiplier suitable for usage in IoT devices with limited resources. The proposed multiplier structure is simple to implement in VLSI technology due to its modularity and regularity. The suggested structure is derived from a formal and systematic technique for mapping regular iterative algorithms onto processor arrays. The proposed methodology allows for control of the processor array workload and the workload of each processing element. Managing processor word size allows for control of system latency, area, and consumed energy. The ASIC experimental results indicate that the proposed processor structure reduces area and energy consumption by factors reaching up to 97.7% and 99.2%, respectively.

## 1. Introduction

The Internet of Things (IoT) is a contemporary technology that links a large number of items to the internet, including wearable devices, sensors, smartphones, smart meters, and auto-mobiles [1,2] It offers services and cost-effective solutions in a variety of fields, including healthcare, smart grid, industrial manufacturing, smart cities, business, and smart railway infrastructure [3,4,5].

For most IoT-based systems, privacy and security are the top priorities. They restrict it from being used in the majority of applications. As a result, to defend IoT-based systems, we should use effective and realistic security solutions. To address all of the security flaws, cryptographic protocols should be used at various levels of the IoT paradigm, particularly, at edge devices. Conventional cryptographic algorithms such as Rivest, Shamir, and Adleman (RSA) and Digital Signature Algorithm (DSA) [6] are expensive to execute on most IoT edge devices due to of their restricted processing capability. The Elliptic Curve Cryptographic (EEC) algorithm [6,7] is the preferred cryptography for resource-constrained integrated devices due to its small key sizes and increased computing effectiveness. The critical part in implementing ECC is the efficient implementation of the finite field multiplication operation. This operation is the core operation in all field arithmetic operations used in ECC such as finite-field inversion and division [8,9,10,11].

### 1.1. Related Work

Depending on the application, finite field multipliers can be built in serial or parallel. When the multiplier is constructed in parallel, it generates all output bits in a single clock cycle, resulting in a significant throughput at the cost of a lot of hardware resources [12,13]. Serial architectures, on the other hand, are optimized for low-space applications at the cost of increasing processing latency to *n* clock cycles, where *n* is the field size [14,15]. We will focus on serial development of the finite field multiplier algorithm because we are targeting resource-constrained IoT applications [15]. The multiplier can be implemented in either a bit-serial or a word-serial fashion. The word-serial version is more economical for resource-constrained IoT devices, because it achieves better area and time complexity than the bit-serial version [16].

The basic four constructions of word-serial finite field multipliers are: serial-in/serial-out (SISO), serial-in/parallel-out (SIPO), parallel-in/serial-out (PISO), and scalable constructions. References [17,18,19,20,21] discussed the polynomial SISO multipliers. The multipliers presented in [17,18,19] have systolic structures that have area complexity of order O(nl) and latecny of order $O(\left\lceil \frac{n}{l}\right\rceil )$, where *n* represents the field size and *l* is the bus word size. The multiplier design proposed in [20] is also a systolic design, but has area complexity of approximately $O(n\sqrt{nl})$ and a lower latency of order $O(2\sqrt{\frac{n}{l}})$. The multiplier design explained in [21] is a three operand non-systolic multiplier with area complexity of order O(nl) and latency of order $O(\left\lceil \frac{n}{l}\right\rceil +2)$.

References [22,23] provide the details of the polynomial SIPO multipliers. The multiplier offered in [22] has a systolic structure with area complexity of order $O(ln\sqrt{\frac{n}{l}})$ and latency of order $O(2\sqrt{\frac{n}{l}})$. The multiplier discussed in [23] has a systolic structure with area complexity of order O(2ln) and latency of order $O(\left\lceil \frac{n}{l}\right\rceil )+l$). In [24], the PISO multiplier was explained using a Type-T Gaussian normal basis. The proposed architecure consumes area of order O(2ln) and has latency of order O(l), but have a very long critical pass delay that it is a function of word size *l*, $O(\log_{2}\left(l\right))$, making the total computation time very high specially for long word sizes.

Later, in [25,26,27,28], the scalable multiplier constructions were discussed in detail. The scalable multipliers of [25,26] are based on a fixed bit-parallel Hankel matrix-vector multiplier whose latency is $\left(l+\left\lceil \frac{n}{l}\right\rceil \left(\left\lceil \frac{n}{l}\right\rceil -1\right)\right)$ clock cycles. The multiplier architecture of [25] has area complexity of order $O(n^{2})$, while the multiplier architecture of [26] has lower area complexity of order $O(l^{2})$. The multiplier of [27] is based on the dual basis multiplication and targets lightweight cryptographic architectures. It has estimated area complexity of order O(n) and latency of order $O(n\left\lceil \frac{n}{l}\right\rceil )$. The design proposed in [28] is a unified structure that performs both multiplication and inversion operations. It has estimated area complexity of order $O(l\left\lceil \frac{n}{l}\right\rceil )$ and latency of the same order.

From the previous discussion, we notice that most SISO multiplier constructions provide improved area and time complexity than other forms of word-serial multiplier constructions. As a result, we will concentrate on obtaining the SISO construction of the adopted algorithm.

### 1.2. Paper Contribution

In this paper, we present a SISO finite field multiplier processor that is two-dimensional (2-D) and word-based. Regularity, modularity, concurrency, and local interconnectivity of the explored processor’s systolic structure are all special aspects, which makes it more convenient for VLSI implementation. The system developer can manage the area and power consumption of the investigated multiplier construction to suit IoT devices by using the formal mapping technique provided in [29,30,31]. The system developer can adjust the workload of the processor array as well as the workload of each processing element by using a non-linear scheduling function. Furthermore, non-linear task scheduling is used to manage the algorithm’s latency. The actual results reveal that the improved multiplier construction saves a large amount of space and energy, making it more suitable for IoT devices with restricted resources.

### 1.3. Paper Organization

The following describes the layout of the manuscript: Section 2 modifies the adopted finite field multiplication algorithm, offered by [32], to be represented in the bit level form. The algorithm performs the multiplication operation over GF($2^{n}$) and is based on the irreducible All-One Polynomial (AOP). The dependency graph (DG) of the explained algorithm is investigated in Section 3. The systematic technique utilized to extract the 2-D word-based SISO processor is explained in Section 4. The experimental findings and analysis of the produced word-based multiplier construction and the competitor ones are displayed in Section 5. Finally, under Section 6, you can find the conclusion of this work.

## 2. Formulation of the Multiplication Algorithm

Suppose that a degree *n* irreducible polynomial U(w) characterizes the finite field over $GF(2^{n})$. It can be described in the polynomial form as:

(1) $$\begin{array}{c} U\left(w\right)=1+u_{1}w^{1}+\cdots +u_{i}w^{i}+\cdots +u_{n-1}w^{n-1}+w^{n} \end{array}$$

with $u_{i}\in GF\left(2\right)$. Consider also that the above polynomial has a root denoted as ζ. As a result, the field elements can be defined by the set of polynomial basis $\left\{1,\zeta ,\zeta^{2},\zeta^{3},\cdots ,\zeta^{n-1}\right\}$.

Assume that polynomials *E* and *H* denote any two field elements in $GF(2^{n})$ space. They can be described in degree n−1 polynomial form as follows:

(2) $$\begin{array}{c} E=e_{0}+e_{1}\zeta^{1}+\cdots +e_{i}\zeta^{i}+\cdots ++e_{n-1}\zeta^{n-1} \end{array}$$

(3) $$\begin{array}{c} H=h_{0}+h_{1}\zeta^{1}+\cdots +h_{i}\zeta^{i}+\cdots ++h_{n-1}\zeta^{n-1} \end{array}$$

where $e_{i},h_{i}\in GF\left(2\right)$.

To multiply *E* and *H* over $GF(2^{n})$, we can use the following formula.

(4) $$\begin{array}{ccc} D & = & E\cdot H\;\mathrm{mod}\;U(w) \end{array}$$

Equation (4) could be extended to include a multiplication recurrence formula as follows:

(5) $$\begin{array}{c} D=h_{0}\cdot E+\left[\sum_{i=1}^{n-1}h_{i}\cdot \zeta^{i-1}\cdot K\right]\;\mathrm{mod}\;U\left(w\right) \end{array}$$

where K=ζE is a polynomial of degree *n* that can be written as:

(6) $$\begin{array}{c} K=\sum_{i=0}^{n}k_{i}\cdot \zeta^{i} \end{array}$$

with $k_{0}=0$ and $k_{i}=e_{i-1}$ for i=1,2,⋯,n.

We can derive the following expression by extending the polynomial of (6) and multiplying by ζ.

(7) $$\begin{array}{c} \zeta K=k_{0}\zeta +k_{1}\zeta^{2}+\cdots +k_{n-1}\zeta^{n}+k_{n}\zeta^{n+1} \end{array}$$

As we mentioned before, ζ is a root of U(w) and this leads to U(ζ)=0. As a result, we can find the following expression by substituting with ζ in Equation (1).

(8) $$\begin{array}{c} \zeta^{n}=1+u_{1}\zeta +u_{2}\zeta^{2}+\cdots +u_{n-1}\zeta^{n-1} \end{array}$$

As U(w) is an AOP polynomial, Equation (8) can be expressed as:

(9) $$\begin{array}{c} \zeta^{n}=1+\zeta +\zeta^{2}+\cdots +\zeta^{n-1} \end{array}$$

By multiplying both sides of Equation (9) by ζ, we obtain the following result:

(10) $$\begin{array}{c} \zeta^{n+1}=1 \end{array}$$

By substituting from (10) in (7), we may reduce ζK to a polynomial ($K^{1}$) of degree *n* as:

(11) $$\begin{array}{c} K^{1}=k_{n}+k_{0}\zeta +k_{1}\zeta^{2}+\cdots +k_{n-1}\zeta^{n} \end{array}$$

As indicated in Equation (11), the cyclic-shift-left of polynomial *K* creates the partially-reduced polynomial $K^{1}$ of polynomial ζK. Additionally, the cyclic-shift-left of polynomial $K^{1}$ produces the partially-reduced polynomial $K^{2}$ of polynomial $\zeta^{2}K$. In general, cyclic-shift-left of polynomial $K^{i-1}$ forms the partially-reduced polynomial $K^{i}$ of polynomial $\zeta^{i}K$. The following is a mathematical representation of the cyclic-shift-left procedure:

(12) $$\begin{array}{c} K^{i}=L\left(K^{i-1}\right),\;\;0\leq i\leq n-1 \end{array}$$

where $K^{-1}=\left(0\&E\right)$. *L* denotes the cyclic-shift-left operation. Equation (12) could be used to construct Equation (13) as:

(13) $$\begin{array}{c} D=h_{0}\cdot E+\left[\sum_{i=1}^{n-1}h_{i}\cdot K^{i-1}\right]\;\mathrm{mod}\;U\left(w\right) \end{array}$$

with $K^{0}=K=\zeta E$.

Alternatively, Equation (13) might be written as:

(14) $$\begin{array}{c} D=V\;\mathrm{mod}\;U(w) \end{array}$$

where *V* is the sum of polynomials of degree *n* that can be expressed as:

(15) $$\begin{array}{c} V=\sum_{i=0}^{n-1}h_{i}\cdot K^{i-1} \end{array}$$

with $K^{-1}=\left(0\&E\right)$.

Equation (15) can be described in the subsequent form:

(16) $$\begin{array}{c} V=v_{0}+v_{1}\zeta^{1}+v_{2}\zeta^{2}+\cdots +v_{n-1}\zeta^{n-1}+v_{n}\zeta^{n} \end{array}$$

By substituting $\zeta^{n}$ in Equation (16) with the expansion given in Equation (9), we could derive the reduced form of polynomial VmodU(w) (polynomial of degree n−1) as:

(17) $$\begin{array}{c} D=V\;\mathrm{mod}\;U\left(w\right)=\left(v_{0}⊕v_{n}\right)+\left(v_{1}⊕v_{n}\right)\zeta^{1}\\ +\left(v_{2}⊕v_{n}\right)\zeta^{2}+\cdots +\left(v_{n-1}⊕v_{n}\right)\zeta^{n-1} \end{array}$$

We can describe Equations (12) and (15) in bit-level format as shown in Equations (18) and (19), respectively. The subscript *j* in these equations denotes the bit position in their binary coding.

(18) $$\begin{array}{ccc} k_{j+1}^{i} & = & k_{j}^{i-1}\\ k_{0}^{i} & = & k_{n+1}^{i} \end{array}$$

(19) $$\begin{array}{c} v_{j}^{i}=v_{j}^{i-1}+h_{i}\cdot k_{j}^{i-1} \end{array}$$

with $k_{n}^{-1}=0$, $v_{j}^{-1}=0$, 0≤i≤n−1, and 0 ≤j≤n.

Equation (17) provides the reduced form of the product polynomial *D*, which can be interpreted in the bit-level formate as:

(20) $$\begin{array}{c} d_{j}=v_{j}^{n-1}+v_{n}^{n-1} \end{array}$$

with 0 ≤j≤n−1.

Algorithms 1 and 2 are the algorithm structure of the previously stated formulas. Algorithm 2 represents the bit-level version of Algorithm 1.
**Algorithm 1** Finite Field Multiplication Algorithm based on AOP polynomial.**Input**: *E*, *H*, and *U***Output**: *D***Initialization**:$K^{-1}\leftarrow \left(0\&E\right)$, $V^{-1}\leftarrow 0$**Algorithm**:1:**for** 0≤i≤n−1 **do**2:    $K^{i}=\beta .\;K^{i-1}$3:    $V^{i}=V^{i-1}+h_{i}K^{i-1}$4:**end for**5:D=VmodU

**Algorithm 2** Finite Field Multiplication Algorithm in the bit-level formate.
**Input**: $E=(0e_{n-1}e_{n-2}\cdots e_{0})$, $H=(h_{n-1}h_{n-2}\cdots h_{0})$
**Output**: $D=(d_{n-1}d_{n-2}\cdots d_{0})$
**Initialization**:


$K^{-1}=\left(k_{n}^{-1}k_{n-1}^{-1}\cdots k_{0}^{-1}\right)\leftarrow \left(0e_{n-1}\cdots e_{0}\right)$




$V^{-1}=\left(v_{n}^{0}v_{n-1}^{0}\cdots v_{1}^{0}v_{0}^{0}\right)\leftarrow \left(00\cdots 00\right)$


**Algorithm**:
1:**for** 0≤i≤n−1 **do**2:    **for** 0≤j≤n **do**3:        $k_{j+1}^{i}=k_{j}^{i-1}$4:        $k_{0}^{i}=k_{n+1}^{i}$5:        $v_{j}^{i}=v_{j}^{i-1}+h_{i}k_{j}^{i-1}$6:    **end for**7:
**end for**
8:**for** 0≤j≤n−1 **do**9:    $d_{j}=v_{j}^{n-1}+v_{n}^{n-1}$10:
**end for**



## 3. Construction of Algorithm Dependence Graph

**Figure 1:**
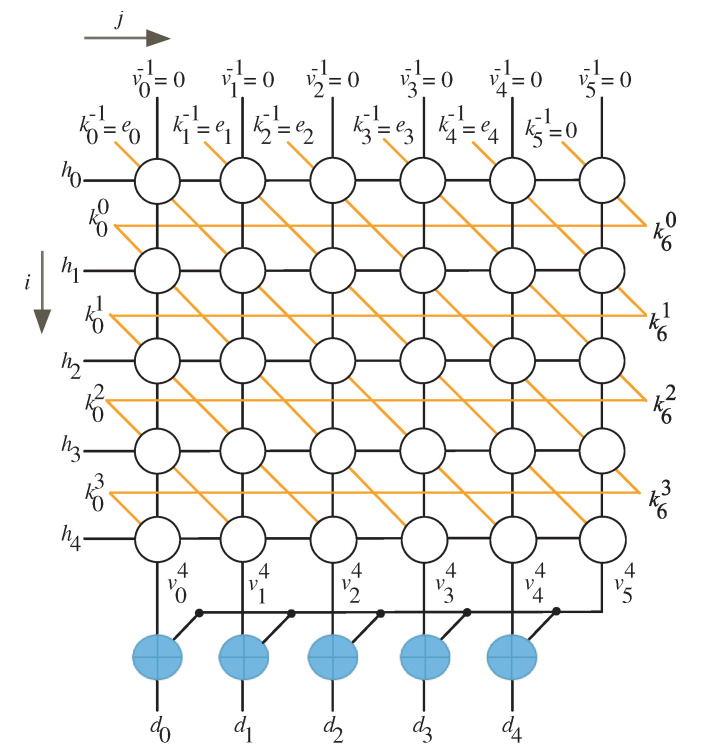
DG of the recommended multiplication algorithm for n=5.

Algorithm 2 has two indices, *i* and *j*, that define the iterative phase of the multiplication algorithm. The approach described in reference [29] can be used to generate a dependence graph (DG) in the two-dimensional integer domain D. Figure 1 shows the DG for the situation when n=5. The nodes of the DG indicates the operations specified by the algorithm steps 3 to 5. According to the design criteria of reference [29], $v_{j}^{i}$ signals are indicated by vertical lines. The $h_{i}$ signals are denoted by horizontal lines. The signals $k_{j+1}^{i}$ are depicted by the diagonal lines.

The signals of $k_{n+1}^{i}$ are generated by the nodes in the last column and transmitted to the nodes in the first column. As indicated in the reduction step of the Algorithm 2, step 9, the resultant signals $v_{j}^{n-1}$, 0≤j≤n−1, are combined with the most significant signal $v_{n}^{n-1}$, using the XOR gates, to generate the final product output signals $d_{j}$, 0≤j≤n−1. The algorithm inputs $v_{j}^{-1}$ and $k_{j}^{-1}=e_{j}$ are displayed in the DG as vertical and diagonal inputs to the top row nodes, respectively. On the other hand, the reduced product output $d_{j}$, 0≤j≤n−1 is created by merging the vertical outputs of the bottom nodes with the output of the most right bottom node as depicted in Figure 1.

Using the technique outlined in [29], the DG of Figure 1 can be used for design space exploration by selecting proper node scheduling and projection functions.

We will not employ the linear scheduling and projection functions presented in [29], as they give us few alternatives for determining the resulting processor array area, latency, processing element workload, and total system workload. We will apply the non-linear node scheduling and projection techniques described in [29] to the DG. This option provides a wide range of design alternatives for optimizing the resulting processor array area, latency, workload of processing elements, and overall system workload.

## 4. Two-Dimensional SISO Multiplier

Our objective is to create a SISO multiplier that accepts inputs *K* and *H* in a word-serial format. In addition, the resultant output *D* is generated from the SISO multiplier in the word-serial format. Assume the system designer’s aim is to process *l* bits of each input at the same time in order to find *l* bits of the output. The following subsections describe the steps that the system developer should follow to construct the SISO multiplier.

### 4.1. Non-Linear Task Scheduling

As explained in [29], the nonlinear scheduling technique is employed to divide the domain D into l×l equitemporal zones or clusters. The *l* value allows the system designer to set the number of bits of inputs and outputs that are processed at the same time. This has an indirect impact on the system’s size, speed, and latency.

To assign timing to each node p of the DG, we use the following non-linear scheduling function:

(21) $$\begin{array}{ccc} k(p) & = & \left\lceil \frac{n}{l}\right\rceil \;\left\lfloor \frac{i}{l}\right\rfloor \;+\left\lfloor \frac{n-1-j}{l}\right\rfloor +1 \end{array}$$

where k(p) is the time allocated to the DG’s node p; 0≤i<n+θ, −θ+1≤j<n−1, and θ is defined as:

(22) $$\theta =l\left\lceil \frac{n}{l}\right\rceil -n$$

**Figure 2:**
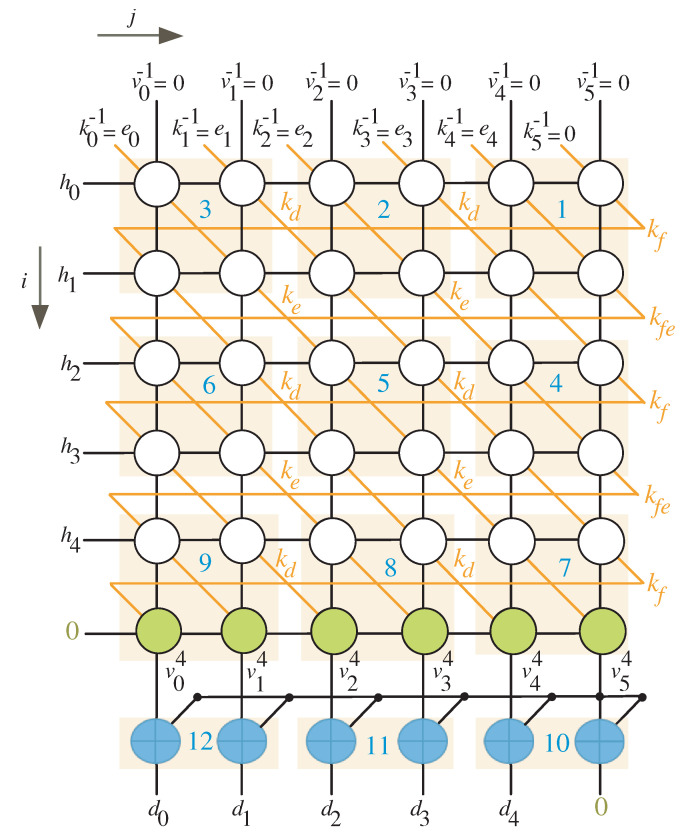
Scheduling time for n=5 and l=2.

To make the DG’s rows an integer multiple of *l*, we should add θ rows to it. In addition, θ−1 columns must be added to the DG in order for the number of columns to be an integer multiple of *l*. We have θ equal to 1 in the scenario depicted in Figure 2 where n=5 and l=2, implying that one row should be placed at the bottom (row with green nodes) and no columns at the left. The equitemporal zones (the cluster of nodes having the same time values) are determined by the light red boxes and marked with the blue numbers as displayed in Figure 2.

**Figure 3:**
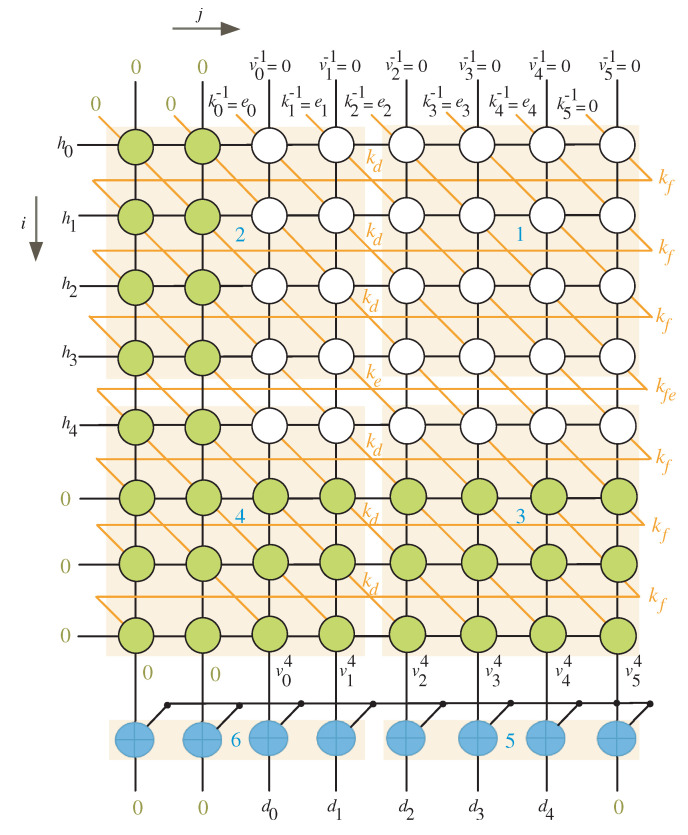
Scheduling time for n=5 and l=4.

The scheduling time for the DG nodes when n=5 and l=4 is shown in Figure 3. We have θ equal to 3 in this scenario, which means we need to employ two columns on the left and three rows on the bottom (rows and columns with green nodes).

By inspecting Figure 2 and Figure 3, we notice that any equitemporal zone (give it name block *k*) takes inputs from the north and west sides and generates outputs from the south and east sides. Table 1 summarizes the timings associated with these inputs and outputs (I/Os).

It is worth noting that the top row’s inputs result in the right column’s outputs. Similarly, the left column’s inputs result in the bottom row’s outputs. As a result, the total number of iterations (I) for finite field multiplication should be calculated using the following expression.

(23) $$I={\left\lceil \frac{n}{l}\right\rceil }^{2}+\left\lceil \frac{n}{l}\right\rceil +1$$

### 4.2. Non-Linear Task Projection

As we observe from Figure 2 and Figure 3, the l×l equitemporal zones execute at the same time. This remark, together with the projection technique described in [29], yields the nonlinear task projection function shown below:

(24) $$\begin{array}{ccc} \bar{p}\left(a,b\right) & = & \left[\begin{array}{cc} i\;\mathrm{mod}\;l & j\;\mathrm{mod}\;l\; \end{array}\right] \end{array}$$

**Figure 4:**
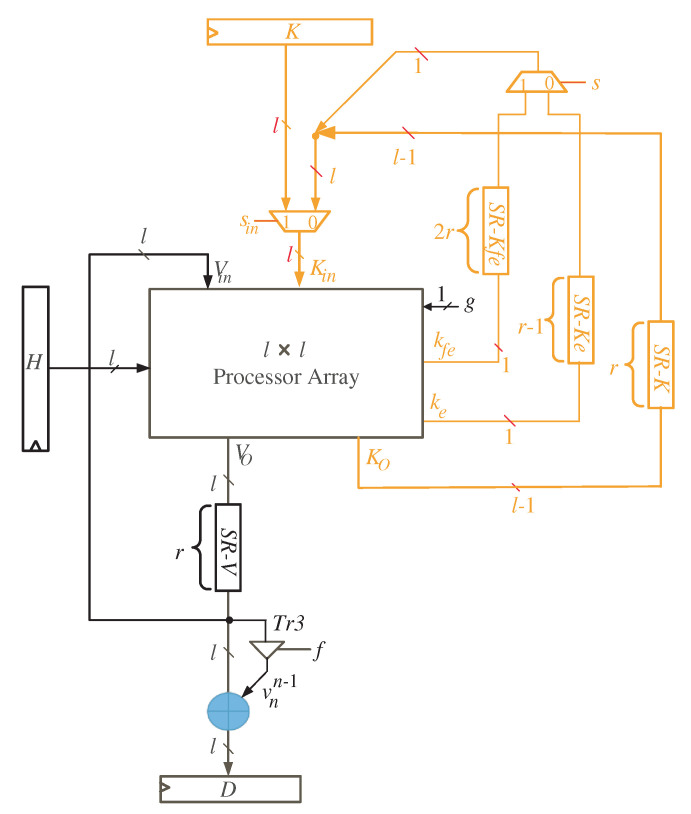
Multiplier SISO processor Structure.

The l×l node clusters are mapped to a single processor array using the extracted projection function. The processor array is made up of l×l processing elements (PEs) that are arranged in a two-dimensional array. The processor structure of Figure 4 depicts the entire system.

By reading Figure 4, we notice that registers *K* and *H* are of size *l* and used to feed the word inputs of *K* and *H*, in sequence, to the processor array block. Furthermore, register *D* is used to synchronize the output product *D* before delivering it to the processor data bus. As input words of variable *V* have zero initial values, there is no need to feed them to the processor array through an input register. They will be initialized by clearing the shift register SR-V shown in the figure. This shift register has a width of *l* bits and depth of *r* registers, where $r=\left\lceil \frac{n}{l}\right\rceil$. The depth of SR-V is sufficient to the guarantee that all the initial input words of variable *V* are fed to the processor array block.

With a closer look at Figure 4, we can notice that the words of *K* variable ($K_{o}$) resulted from the processor array block have three different types of signals due to the delay differences between signals $K_{e}$, $K_{fe}$, and the remaining signals of word *K*, as shown in Figure 2 and Figure 3. $K_{e}$ signal should be delayed by r−1 time steps, $r=\left\lceil \frac{n}{l}\right\rceil$, before feeding it back to the input of the processor array block. Additionally, before returning the $K_{fe}$ signal to the input of the processor array block, it should be delayed by 2r time steps. The remaining signals of the word *K* ($K_{o}$) should be delayed by *r* time steps before being fed back to the processor array’s input. These delays are implemented using the shift registers (SR) related to variable *K* as shown in Figure 4. The width and depth of each SR are indicated in the figure. As we also notice from Figure 4, the intermediate words of *V* are looped back through the shift register SR-V to be delayed by *r* time steps before reaching out to the inputs of the processor array block.

**Figure 5:**
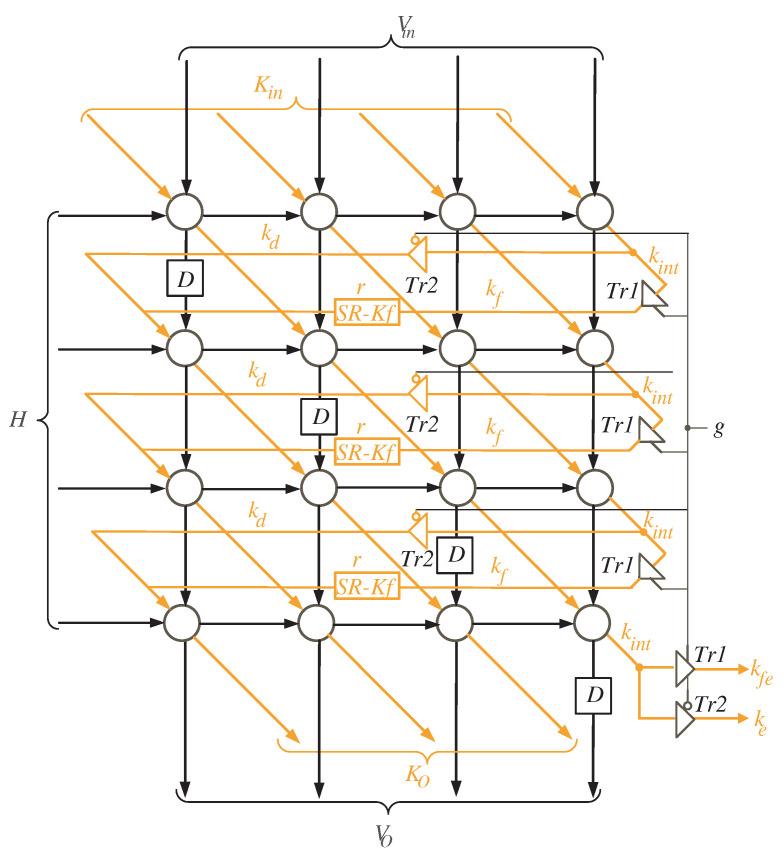
The structure of Multiplier SISO processor array.

The processor array description is shown in Figure 5 for the case when n=5 and l=4. Two types of tri-state buffers are used to select between signals $k_{d}$ and $k_{f}$. Another two types of tri-state buffers are used to select between signals $k_{e}$ and $k_{fe}$. All of these buffers are controlled with the control signal *g*. At time instances k=q⌈n/l⌉+1, 0≤q<⌈n/l⌉, the control signal *g* is enabled (g=1), allowing the tri-state buffers Tr1 to pass $k_{f}$ and $k_{fe}$ signals shown in Figure 5. The control signal *g* is deactivated (g=0) for the remaining time instances, allowing the $k_{d}$ and $k_{e}$ signals to pass through tri-state buffers Tr2.

**Figure 6:**
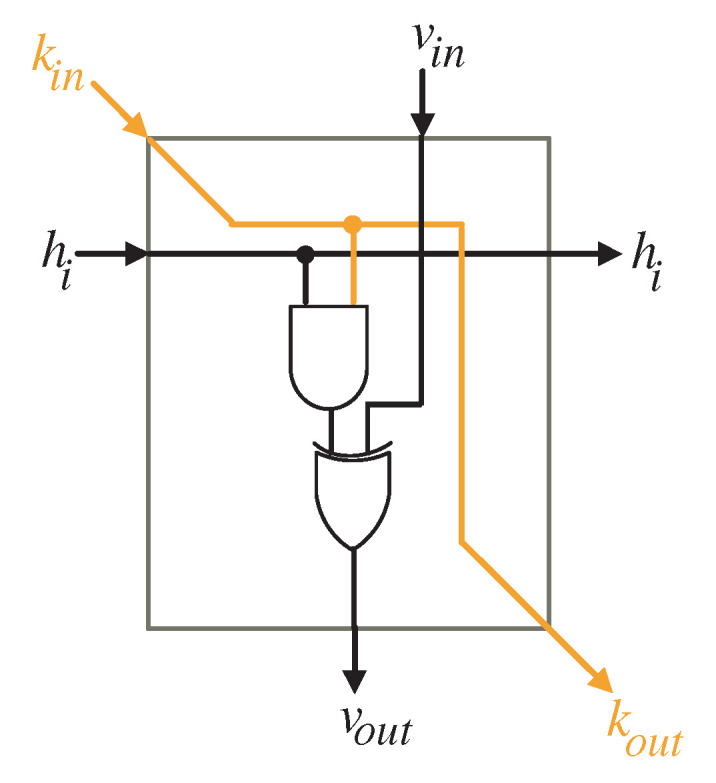
PE logic details.

To compute the intermediate bits of word *V*, the input bits of word *H* ($h_{i}$) should be transferred to the processing elements of the processor array as displayed in Figure 5. The logic diagram of the PE is depicted in Figure 6. It includes one AND gate and one XOR gates.

The operation details of the 2-D SISO multiplier for general values of *n* and *l* are as follows:

1. At the first time instance k=1, the controller activates the MUX with select signal ($S_{in}$) to allow the *l* most significant bits (MSB) of variable *K* to reach out to the input of the processor array block as shown in Figure 4. To ensure *V* variable has zero initial value as described in Algorithm 1, the controller resets the shift register SR-V at the first time instance. At the same time instance, the least significant *l* bits of variable *H* are transmitted horizontally to the PEs nodes of the processor array block. Notice that the *H* word transferred at this time instance should be hold for the following $\left\lceil \frac{n}{l}\right\rceil -1$ time instances.
2. At time instances $1<k\leq \left\lceil \frac{n}{l}\right\rceil$, the controller still activates the MUX with select signal ($S_{in}$) to enable the remaining words of input *K* to reach out to the processor array input. These words, together with the previously held *H* words at the first time instance, are used to calculate in sequence the partial words of *V* and *K*. The *V* words resulted from the output of the processor array block ($V_{o}$) are looped back to its input through the shift register SR-V. The *K* words resulted from the output of the processor array block are looped back to its input through the shift registers SR-*K*, SR-*Ke*, SR-*Kfe*, and the MUX controlled by the select signal *S* as displayed in Figure 4. It is worth noticing that the depth of the shift register SR-V keeps the initial values of *V* having zero values during these time instances.
3. During times $k=q+(\left\lceil \frac{n}{l}\right\rceil -1)$, $2\leq q\leq 2\lceil \frac{n}{l}\rceil$ and $q\neq \lceil \frac{n}{l}\rceil +1$, the controller deactivates the MUX controlled by the select signal *S* (S=0), see Figure 4, to pass the $K_{e}$ signal to be concatenated with the $K_{o}$ word. At the same time instances, the controller deactivates the MUX controlled by the select signal $S_{in}$ ($S_{in}=0$) to transfer the whole partial word of *K* to the input of the processor array block as displayed in Figure 4.
4. During times $k=\left(q+1\right)\left\lceil \frac{n}{l}\right\rceil$, $1\leq q<\lceil \frac{n}{l}\rceil$, the controller activates the MUX controlled by the select signal *S* (S=1), see Figure 4, to pass the $K_{fe}$ signal to be concatenated with the $K_{o}$ word. At the same time instances, the controller deactivates the MUX controlled by the select signal $S_{in}$ ($S_{in}=0$) to transfer the whole partial word of *K* to the input of the processor array block as displayed in Figure 4.
5. At times $k=q\lceil \frac{n}{l}\rceil +1$, $0\leq q<\lceil \frac{n}{l}\rceil$, the remaining *H* words are transferred to the input of the processor array block to be used alongside the word inputs $V_{in}$, $K_{in}$ in updating the partial words of variable *V* ($V_{o}$).
6. At time $k=\left(\left\lceil \frac{n}{l}\right\rceil -1\right)\left\lceil \frac{n}{l}\right\rceil +1$, the control signal *f* of the tri-state buffer Tr3, shown in Figure 4, is set (f=1) to pass the signal $v_{n}^{n-1}$ to be XORed with the words of *V* to find the output product words *D*, in sequence, as displayed in Figure 4.
7. Starting at time $k={\left\lceil \frac{n}{l}\right\rceil }^{2}+1$, the output words of product *D* will be available in sequence at the output bus.

To ensure that there is always one time instance difference between the words of *V*, we inserted delay elements (D Flip-Flop blocks) to the processor array, as illustrated in Figure 5. These elements synchronize the processor array’s work by delaying *V* words by one time instance to arrive at the same time as the resultant bits of $k_{d}$. The $k_{d}$ bits are created starting at the second time instance, as seen in Figure 3, and this results in increasing the total number of clock cycles by one as indicated in Equation (23). Furthermore, shift registers SR-*Kf* of depth *r* are added to the processor array (see Figure 5) to ensure that the $k_{f}$ signals arrive at the left processing elements at the appropriate time.

## 5. Experimental Results and Discussion

We compared the suggested 2-D word-based multiplier structure to the optimal word-based ones in the literature [20,23,33,34]. The area estimation is determined by the number of basic logic gates and components in the examined multiplier architectures (AND gates, Tri-state buffers, XOR gates, Flip-Flops (FFs), and MUXs). The number of clock cycles needed to accomplish the multiplication operation is defined as latency. The delay of the basic gates/components of the multiplier logic circuit’s longest path is referred to as critical path delay (CPD). The estimated area and time results of the multiplier structures are shown in Table 2. The following symbols are used in Table 2. They can be translated as follows:

∎ *l* denotes the word size of the multiplier constructions.
∎ $\delta_{A}$ denotes the delay of the fundamental 2-input AND gate.
∎ $\delta_{X}$ denotes the delay of the fundamental 2-input XOR gate.
∎ $\delta_{MUX}$ denotes the delay of the 2-input MUX.
∎ $\alpha_{1}=7n+n\left(\left\lceil \log n\right\rceil \right)+l+3$ expresses the overall number of FFs employed in the multiplier construction of Pan [20].
∎ $\alpha_{2}=2l^{2}+2l\left(\left\lceil n/l\right\rceil \right)+4l+1$ expresses the overall number of FFs employed in the multiplier construction of Hua [33].
∎ $\alpha_{3}=2l^{2}+3l\left(\left\lceil n/l\right\rceil \right)+2l$ expresses the overall number of FFs employed in the multiplier construction of Chen [34].
∎ $\eta_{1}=l+{\left\lceil n/l\right\rceil }^{2}+\left\lceil n/l\right\rceil$ designates the latency of the multiplier construction of Chen [34].
∎ $\beta_{1}=\delta_{A}+\left(\left\lceil \log_{2}l\right\rceil +1\right)\delta_{X}$ is the approximated CPD of Pan’s multiplier construction [20].
∎ $\beta_{2}=\delta_{A}+2\delta_{X}$ is the approximated CPD of Hua’s multiplier construction [33].
∎ $\beta_{3}=\delta_{A}+\delta_{X}$ is the approximated CPD of Chen’s multiplier construction [34].
∎ $\beta_{4}=l\delta_{A}+l\delta_{X}+2\delta_{MUX}$ is the approximated CPD of the suggested multiplier construction.

It is worth mentioning that the input/output registers are included in the approximated number of FFs. This guarantees that the multiplier architectures are fairly compared.

We can find the following conclusions from examining the area expressions in Table 2:

1. The area complexities of Pan [20] and Xie [23] multipliers are roughly of order $O(n\sqrt{nl})$ and O(nl), respectively.
2. Except for the MUXes and FFs of the recommended multiplier structure, which have area complexity of order O(l) and O(lr), all other components have area complexity of order $O(l^{2})$.
3. Pan’s [20] and Xie’s [23] multiplier constructions have a larger area complexity than the other multiplier constructions. This is due to the fact that the field size *n* is significantly bigger than the embedded word size *l*.
4. In comparison to the other multipliers, the suggested multiplier has the smallest number of FFs. This is due to the suggested multiplier having an area complexity of order O(lr), as opposed to $O(l^{2})$ and O(n(⌈logn⌉) for the other multiplier structures.
5. The number of FFs in the proposed multiplier structure does not rise significantly as the word size *l* is increased. This is due to the fact that the proposed multiplier structure’s FFs have an area complexity of order O(lr).

According to the data books of most typical CMOS libraries, the FFs require more chip space than the other logic components. As a result, lowering the number of FFs reduces the overall size of the multiplier structures dramatically. Increasing the word size does not considerably increase the overall number of FFs in the proposed multiplier structures, as we previously stated. As a result, the overall area of the suggested multiplier structure will not rise considerably as *l* grows.

We can notice the following by examining the latency expressions in Table 2:

1. When compared to the other multiplier constructions, the multiplier of Hua [33] has the lowest latency.
2. The latency findings in Table 3, for the field size n=508 and word sizes l=8,16,32, indicate that the suggested multiplier structure’s latency expression will result in a larger latency than the multiplier constructions in [20,23], and inexpensive latency compared to the Hua [33] and Chen [34] multiplier constructions.
3. When the word size *l* increases, the latency reduces. This is due to the fact that latency expressions are inversely related to *l*.

We could remark the following facts when we examine CPD expressions:

1. The word sizes *l* have no effect on the CPD expressions of the Xie [23], Hua [33], and Chen [34] multiplier constructions. As a result, for all *l* values, they will always have constant CPD values.
2. CPD expressions of Pan [20] and the proposed multiplier structure are both directly dependent on *l*. As a result, the CPD values of these multipliers will rise as *l* rises.

We cannot accurately predict which multiplier architecture has the perfect computation time because it is challenging to qualitatively evaluate the latency reduction and CPD increment as *l* rises. Nevertheless, the quantitative results provided in Table 3 will demonstrate which multiplier layout outperforms the others in computation time.

The VHDL programming language has been used to describe all of the multiplier constructions. For the field size n=508 and embedded word sizes l=8,16,32, the multipliers are synthesized. Synopsys tools version 2005.09-SP2 and the NanGate (15 nm, 0.8 V) Open Cell Library have been used to synthesize the modeled multipliers.

The design performance indicators—Latency, Area (A), CPD, Total Computation Time (T), Consumed Power (P), and Consumed Energy (E)—are used to compare the chosen word-based multiplier constructions. The obtained results are listed in Table 3. The area and CPD are provided by the synthesis tools. The area of a 2-input NAND gate is used to normalize the area. The needed time to accomplish one product operation can be defined as the total computation time. It is calculated by multiplying latency and CPD together. At a frequency of 1 kHz, the consumed power is measured. The product of P and T yields the consumed energy results.

The performance results achieved in Table 3 can be interpreted as follows:

1. In terms of area (A), the proposed multiplier structure is superior to all existing multiplier structures. It greatly decreases area for all embedded word sizes *l*, with reduction rates ranging from 67.3% to 97.7%. The reduction in area is primarily due to the proposed multiplier structure’s area, which is mainly determined by the field size *l*, drastically reducing the number of counted logic gates when compared to most other existing multiplier structures. Furthermore, due to the systolic nature of the suggested multiplier, the majority of its connections are local, leading to a reduction in the area to a large extent.
2. In terms of the area-time product (AT), Pan’s multiplier structure [20] surpasses all other multiplier structures, including the suggested one, at l=8. This is mainly attributed to the significant reduction in its latency compared to the other multiplier constructions at this word size. At this embedded size, it outperforms the offered design by 37.9%. The proposed architecture, on the other hand, surpasses Pan’s multiplier structure for l=16 and l=32. At l=16, it reduces AT by 26.3%, while at l=32, it reduces AT by 49.2%. Furthermore, the suggested multiplier structure outperforms all alternative multiplier structures by percentages ranging from 21.1% to 99.4% based on the embedded word size.The reduction in AT over the other multiplier structures is mainly due to the significant savings in area complexity of the suggested multiplier structure.
3. In terms of consumed power (P), the proposed multiplier outperforms the other multiplier structures at all embedded word sizes. It reduces power consumption at all *l* values by percentages ranging from 64.4% to 99.5%. The power reduction is attributed to the substantial reduction in the consumed area of the proposed design when compared to the consumed area of the other multiplier designs. The reduced area minimises parasitic capacitance and, as a result, the circuit’s dynamic power significantly reduces. The systolic nature of the proposed design reduces the switching activities of the proposed design compared to the other conventional designs. The switching activities is one of the major parameters that significantly affects the dynamic power consumption.
4. In terms of consumed energy (E), the offered multiplier construction surpasses the other multiplier constructions at all embedded sizes. It saves energy at rates ranging from 70.6% to 99.2%. The energy savings are due to the massive reduction in consumed power and the reasonable computation time of the offered multiplier construction compared to the other multiplier structures.

From the obtained results, we can conclude that the offered multiplier outperforms its competitors in terms of area, consumed power, and consumed energy for all popular embedded word sizes. As a result, the proposed design can be used to efficiently implement crypto-processors in resource-constrained IoT devices such as wearable and implantable devices. It can also be used in other resource-constrained applications that set restrictions on the area and energy consumed.

## 6. Summary and Conclusions

In this paper, we offered a compact and practical 2-D word-based serial-in/serial-out processor for the finite field multiplier in GF($2^{n}$). A rigorous and systematic technique for mapping regular iterative algorithms onto processor arrays is used to create the proposed processor structure. The methodology enables the system developer to manage the overall workload of the processor array system as well as the workload of each processing element. Controlling processor word size allows us to adjust system speed, latency, and area. The recommended processor size can be adjusted to meet the intended chip area, allowing for better implementation of the suggested multiplier processor in resource-constrained IoT devices. The obtained experimental results confirm that the suggested multiplier processor has the benefit of reducing size, power consumption, and utilized energy when compared to the conventional multiplier processor.

## 7. Future Work

As a future work, we will incorporate the proposed multiplier into the ECC cryptography to evaluate the amount of savings in its area and consumed energy. The process will start by replacing the inversion operation by several multiplication operations by representing the elliptic curve points as projective coordinate points.

## Figures and Tables

**Table 1:** I/Os timing for block *k*.

I/O	Time Instance
North input ($K_{n}$)	*k*
East output ($K_{e}$)	*k*
West input ($K_{w}$)	k+1
South output ($K_{s}$)	k+1

**Table 2:** The word-based multipliers’ area and time complexities.

Design	Tri-State	AND	XOR	MUXs	FFs	Latency	CPD
Xie [23]	0	2nl	$2nl+6n-6\frac{n}{l}+6$	0	4nl+4n+2l	$2r+2\lceil \log_{2}l\rceil$ ${}^{\left(1\right)}$	$2\delta_{X}$
Pan [20]	0	$n\sqrt{n}$	$\sqrt{nl}\left(2+n\right)+l$	0	$\alpha_{1}$	$2\lceil \sqrt{n/l}\rceil$	$\beta_{1}$
Hua [33]	0	$l^{2}$	$l^{2}+4-5l+1$ ${}^{\left(2\right)}$	0	$\alpha_{2}$	6lr	$\beta_{2}$
Chen [34]	0	$l^{2}+l$	$l^{2}+2l$	2l ${}^{\left(3\right)}$	$\alpha_{3}$	$\eta_{1}$	$\beta_{3}$
Proposed	2l+1	$l^{2}$	$l^{2}+l$	l+1	(2l+5)r−1)	$r^{2}+r+1$	$\beta_{4}$

^(1)^$r=\left\lceil \frac{n}{l}\right\rceil$; ^(2)^ The area of a 3-input XOR gate is 1.5 × that of a 2-input XOR gate; ^(3)^ In [34], the multiplier employs switches with the same level of complexity as a MUX.

**Table 3:** Performance results of word-based modular multipliers for n=508 and various embedded word sizes *l*.

Multiplier	*l*	Latency	Area (A)	CPD	Time (T)	Power (P)	Energy (E)	AT	%A	%AT	%P	%E
			(*Kgates*)	(*ps*)	(*ns*)	(*nW*)	(*fJ*)					
	8	386	110.7	67.1	25.9	268.5	7	2866.4	97.3	21.1	99.4	82.9
Xie [23]	16	205	174.9	67.1	13.7	447.4	6.1	2396.5	97.7	43.6	99.4	86.1
	32	117	232.2	67.1	7.8	568.1	4.4	1810.9	97.8	47.3	99.3	84.6
	8	58	115.9	245.5	14	301.1	4.2	1622.7	97.4	−37.9	99.5	72.0
Pan [20]	16	43	147.6	290.8	12.5	380.9	4.8	1844.5	97.3	26.3	99.3	82.3
	32	29	195.5	336.2	9.6	505.9	4.9	1876.9	97.4	49.2	99.2	86.1
	8	308,905	9.5	87.3	26,981.6	5.2	141.3	256,864.8	68.8	99.1	70.5	99.2
Hua [33]	16	154,453	12.4	87.3	13,490.8	7.0	94.7	166,962.1	67.3	99.2	64.4	99.1
	32	77,227	23.8	87.3	6745.4	13.2	89.1	160,540.5	79.0	99.4	71.2	99.2
	8	14,216	12.1	65.7	933.8	6.1	5.7	11,334.5	75.5	80.0	74.5	79.4
Chen [34]	16	4377	16.1	65.7	287.5	9.9	2.9	4618.7	74.8	70.7	75.0	70.6
	32	1871	31.7	65.7	122.9	19.0	2.3	3890.3	84.2	75.5	80.0	71.4
	8	3281	2.9	231.8	760.5	1.547	1.2	2262.5	-	-	-	-
Proposed	16	837	4.0	400.2	334.8	2.5	0.8	1354.6	-	-	-	-
	32	218	4.9	876.1	190.8	3.8	0.7	953.6	-	-	-	-

## Data Availability

Not Applicable.

## Abbreviations

The following abbreviations are used in this manuscript:

IoT	Internet of Things
ASIC	Application Specific Integrated Circuit
ECC	Elliptic Curve Cryptography
DG	Dependency Graph
AOP	All-One Polynomial
VLSI	Very Large Scale Integrated Circuit
L	Cyclic-Shift-Left
DSA	Digital Signature Algorithm
FFs	Flip-Flops
RSA	Rivest, Shamir, and Adleman
SISO	Serial-In/Serial-Out
SIPO	Serial-In/Parallel-Out
PISO	Parallel-In/Serial-Out
CPD	Critical Path Delay
